# How thorough clinical observational studies on rheumatoid arthritis can have an impact on the field

**DOI:** 10.1186/s13075-019-2023-7

**Published:** 2019-11-07

**Authors:** A. H. M. van der Helm-van Mil

**Affiliations:** Department of Rheumatology, Leiden University Medical Center, Leiden, the Netherlands

It is with enormous pleasure that I congratulate *Arthritis Research & Therapy* on their 20th birthday since their launch in 1999. It is a great achievement, especially as the first Open Access journal in the field of rheumatology. We learned that our manuscript “Antibodies to citrullinated proteins and differences in clinical progression of rheumatoid arthritis” of 2005 has ended in the top most cited articles [1]. It puts forward the general question what studies have impact on the field. In addition, with respect to this study, we will look back why this study was performed and how the field has changed since then.

In general, studies that change the field are those that either importantly improve the understanding of disease pathogenesis or have had impact on patients care in daily practice. Such important studies are relatively easily recognized in retrospect, but are less easily recognizable at the time of performance and first reporting, among others because subsequent studies are generally required to validate or expand on the initial findings.

Our study was published in 2005; anti-citrullinated protein antibodies (ACPA) were identified at that time, and routine measurement took place in some but not yet in most clinics. It had also been discovered that these autoantibodies occur very early in the development of rheumatoid arthritis (RA) and were highly specific for the disease, all pointing to the diagnostic potential. However, to what extent patient characteristics, either at disease presentation or progression, differed between patients with and without ACPA was unknown. Taking advantage of RA patients included in the Leiden Early Arthritis Clinic cohort, we observed that the first clinical presentation of both groups of patients was similar, except from a single difference in joints that were reported as the location of the first symptoms (ACPA-positive patients had more often symptoms at upper and lower extremities, and ACPA-negative patients more frequently only upper extremities), a finding that was thought to be possibly false positive. More importantly, we demonstrated that patients with ACPA had a more severe disease course, with higher numbers of inflamed joints and more severe radiographic progression [1]. This finding has contributed to ACPA being seen not only as a diagnostic marker, but also as a prognostic marker.

What has happened since 2005? ACPA testing has become routinely integrated into diagnostic process of RA and has become part of the 2010 classification criteria. More importantly, the radiographic outcome of patients with RA has dramatically improved, due to early initiation of DMARD treatment, treat-to-target treatment adjustments, and the increasing availability of new DMARDs for patients that failed on conventional DMARDs. This improvement is not related to the identification of ACPA as prognostic factor, and current guidelines for initial therapy still do not promote to treat ACPA-positive and ACPA-negative RA differently [2, 3]. Nonetheless, the outcome of patients with RA, and ACPA-positive RA in particular, has been improved to such an extent that results from 2005 can no longer be replicated in radiographic data collected from patients today. In addition to improvements in disease activity and joint destruction, disease outcomes that are most important to the patients themselves (e.g., pain, fatigue, workability) have improved such that traditional differences between ACPA-positive and ACPA-negative patients have disappeared [4]. In other words, with current treatment strategies, the severity of ACPA-positive and ACPA-negative RA has become equal in terms of several outcomes.

The contribution of ACPA to disease pathogenesis was unclear in 2005, and this has not considerably changed since then. It remains questionable if ACPA is causally related to disease development. Arguments against are that ACPA is present in the general population (with a low frequency) without causing symptoms and often without progression to RA. Also, in patients with non-specific musculoskeletal symptoms or with clinically suspect arthralgia, the presence of ACPA is not related to progress to RA in ~ 30–70% of cases [5]. In experimental mice models, passive transfer of monoclonal ACPA has not consistently resulted in development of clinical arthritis. Despite much attention that has been paid to ACPA, further basic and translational studies are required to clarify the pathogenesis of RA.

However, progress has been made since 2005. Genetic studies have identified > 100 genetic risk factors, predisposing almost exclusively for ACPA-positive RA and only a few genetic variants predispose to ACPA-negative RA [6]. Similarly, genetic studies on the severity of radiographic progression have identified genetic markers, several of which predispose to a more severe course of disease in just one of the two subgroups [7]. Also, environmental risk factors were reported to be different between the two groups [8, 9]. Moreover, detailed clinical observational studies that were performed in the symptomatic phase preceding clinical arthritis have revealed several differences in the first symptoms between both groups, among which a difference in initial involvement of upper and lower extremities in ACPA-positive RA and upper extremities in ACPA-negative RA [10]. This finding is in line with our finding done in early RA patients in 2005 [1]. Finally, results from five observational cohorts revealed that ACPA-positive RA develops at a slightly younger age than ACPA-negative RA [11]. Together, these findings provide additional evidence that ACPA-positive and ACPA-negative RA can be considered as separate disease entities.

What can have an impact on the field and on the lives of patients in the near future? Despite the current evidence suggesting that ACPA-positive and ACPA-negative RA are intrinsically different, and although on group level ACPA-positive RA more often needs intensive treatment to achieve DAS remission than ACPA-negative RA, it is still undetermined if treatment of RA should be personalized with respect to the presence of RA-related autoantibodies. In other words, it is unknown if early DMARD start with methotrexate and subsequent treat-to-target is the optimal strategy for both disease subsets. Large clinical studies evaluating long-term disease outcomes are required to determine if ACPA-positive and ACPA-negative RA respond differently to current regular treatment. Findings of such observational studies can fuel trials that aim to identify the optimal treatment strategy for each subgroup separately. Therefore, next to basic studies on RA pathogenesis, observational clinical studies remain relevant to determine whether RA should be subdivided into subgroups relevant for clinical practice.

## Data Availability

Not applicable as no primary data is presented.

## Abbreviations

ACPA: Anti-citrullinated peptide antibodies
RA: Rheumatoid arthritis
